# Effect of Polymer Matrix on the Structure and Electric Properties of Piezoelectric Lead Zirconatetitanate/Polymer Composites

**DOI:** 10.3390/ma10080945

**Published:** 2017-08-14

**Authors:** Rui Li, Jun Zhou, Hujun Liu, Jianzhong Pei

**Affiliations:** 1 Highway School, Chang’an University, Xi’an 710064, China; Herman.2352247@gmail.com (J.Z.); Peijianzhong@126.com (J.P.); 2 School of Materials Science and Engineering, Nanyang Technological University, Singapore 639798, Singapore; 3 CCCC Fourth Harbor Engineering Institute Co., LTD., Guangzhou 510000, China; Jacquelinhomegrow13561@gmail.com

**Keywords:** PZT/polymer, PVDF, PVC, dielectric, piezoelectric

## Abstract

Piezoelectric lead zirconatetitanate (PZT)/polymer composites were prepared by two typical polymer matrixes using the hot-press method. The micromorphology, microstructure, dielectric properties, and piezoelectric properties of the PZT/polymer composites were characterized and investigated. The results showed that when the condition of frequency is 10^3^ Hz, the dielectric and piezoelectric properties of PZT/poly(vinylidene fluoride) were both better than that of PZT/polyvinyl chloride (PVC). When the volume fraction of PZT was 50%, PZT/PVDF prepared by the hot-press method had better comprehensive electric property.

## 1. Introduction

In recent years, the use of piezoelectric materials to collect energy received more and more attention, the environmental characteristics of piezoelectric materials are also gaining popularity [1,2,3,4]. As a member of the new functional material family, piezoelectric actuators have been widely used in engineering and daily life because of their good piezoelectric effect, but their inflexibility and brittleness lead to processing difficulties and poor impact resistance, and so their applications are limited [5,6,7]. In the past, researchers improved the properties of PZT by changing its preparation condition, polarization condition, and the particle size in most cases, but there have been few studies to examine how the polymer affects PZT/PVDF.

Compared with the pure PZT piezoelectric materials, PZT/polymer composites have many virtues such as lower density, better flexibility, stronger plasticity, easier performance improvement and so on. Mendes [8] researched the influence of particle size and the amount of PZT fillers on the PVDF matrix of measurement the dielectric and dynamic mechanical of PZT/PVDF, the results showed that the dielectric properties of the composites are mainly affected by the amount of the ceramic particles. As a polymer monomer, poly(vinylidene fluoride) (PVDF) is renowned for having excellent piezoelectric properties, as well as good mechanical and chemical properties, especially a wide processing temperature range to meet the industrial control system field [9,10,11,12,13,14,15]. As an engineering polymer, polyvinyl chloride (PVC) is widely used throughout the building materials industry because it has unique advantages such as waterproof, fireproof, and antistatic properties, PVC can be especially easily shaped and costs less than PVDF [16,17,18,19,20,21,22,23].

In this paper, PVDF and PVC were used to prepare PZT/polymer piezoelectric composite with PZT respectively, the microstructure of PZT/polymer composite was observed by scanning electron micrographs (SEM), the physical phases of the composite were analyzed by X-ray diffractometer (XRD), the effect of two kinds of polymer matrix on the electric properties of PZT/polymer composite materials were investigated. The results measured have great importance to the design of application domain of PZT/polymer composite materials.

## 2. Experimental

### 2.1. Materials

PZT was supplied by Hongsheng Acoustic, Electronic Equipment Co. Ltd. in Baoding, China. PVDF was supplied by San AI Fu FR902 in Shanghai, China. PVC was from Nanjing Daoning Chemical Co. Ltd. in Nanjing, China.

### 2.2. Preparation of PZT/Polymer Composites

The PZT and PVDF powder were weighed by the volume. Then they were mixed and the mixture was placed into a grinding bowl, next absolute ethyl alcohol was added, the powder was mixed with a stirring rod for 10 min. After the mixture was dried in an oven at 100 °C for 25 min, the mixed powder of PZT and PVDF was put in the hot pressing mold, which was heated to 190 °C for 30 min, PZT/PVDF composites with a diameter of 13 mm and a thickness of 1 mm were obtained.

The above steps were repeated to mix the PZT and PVC, the temperature of the hot pressing mold was 175 °C for 30 min, PZT/PVC composites with a diameter of 13 mm and a thickness of 1 mm were obtained.

The pan was then dried in an oven and an electrode was coated thereon. It was placed in thermostatic silicone oil and polarized at a polarization voltage of 3 kV/mm and at 80 °C for 30 min. Finally, the samples were tested after resting 24 h.

### 2.3. Measurements

All these measurements were obtained at ambient temperature and pressure. The microstructures of PZT/polymer composites were characterized by XRD, FT-IR, and SEM, the dielectric constant (ε_r_) and the dielectric loss (tan δ) of PZT/Polymers composites were measured by a precision LCR digital bridge under 1 kHz, and the piezoelectric constant (d_33_) of PZT/Polymer composites were measured by ZJ-3AN-type quasi-static d_33_ measuring.

## 3. Results and Discussion

### 3.1. Structure and Morphology

#### 3.1.1. XRD

**Figure 1:**
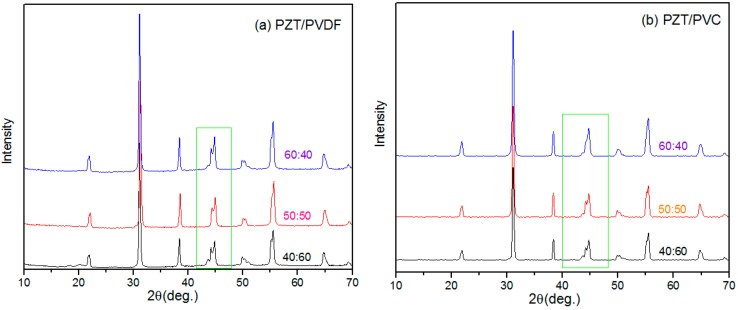
(**a**) XRD pattern of PZT/PVDF composites; (**b**) XRD pattern of PZT/PVC composites.

Figure 1 shows the XRD pattern of PZT/PVDF and PZT/PVC composites. It can be seen that there are no new diffraction peaks of XRD and the diffraction peak of PZT has not changed significantly. It is only because of the different crystallization of the polymer that there are different manifestations in the XRD of composite. PVDF is a semicrystalline polymer whose diffraction peak is weak and is basically covered when the PZT content is 50%. Figure 1b shows that there is no the diffraction peak of PVC caused by the PVC crystallinity is low. with the increase of the PZT content, the (002) diffraction peak becomes gets stronger gradually, that is, with the increase of PZT content and decrease of polymer content, the formation is beneficial to the polarization of the ceramic phase.

It is not hard to find that the figure of PZT/PVDF composite is similar to the PZT/PVC, but the PZT/PVC is slightly wider, but the polarization rate of the PVDF base composite is higher than that of the PVC base composite material at high content. When the content of PVDF is high, the PZT polarization rate of PZT/PVDF composites is higher than that of PZT/PVC composites [24].

#### 3.1.2. FT-IR

**Figure 2:**
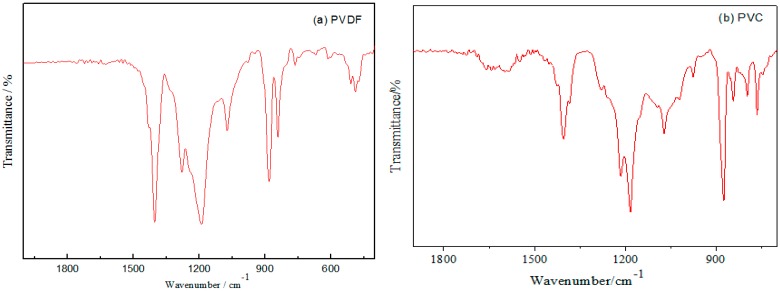
(**a**) FT-IR spectra of PVDF powders; (**b**) FT-IR spectra of PVC powders.

Figure 2a is FT-IR spectra of PVDF powder. It can be seen from the absorption band that the PVDF primarily existed as α-phase, but exhibited only one δ phase absorption band with the wave number of 510 cm^−1^. It means that it is difficult to obtain the β-PVDF with piezoelectric effect through the hot pressing method. For the piezoelectric composites prepared by hot pressing, PVDF is mainly α crystal and does not have the piezoelectric properties. That is, PVDF mainly plays a role of connection in PZT/PVDF composites, and its piezoelectric properties can be neglected. However, due to the strong polarity of C–F in PVDF, the effect of dielectric properties of the PZT/PVDF composites cannot be neglected. Figure 2b shows that the maximum value of the PVC absorption peak at 1248.82 cm^−1^, which caused by the oscillation of the adjacent –C– of the CH_2_ functional group in the PVC and its connected –Cl– atoms. The vibration absorption peak of ethylene at 1429.08 cm^−1^ was formed due to shear distortion.

#### 3.1.3. SEM

**Figure 3:**
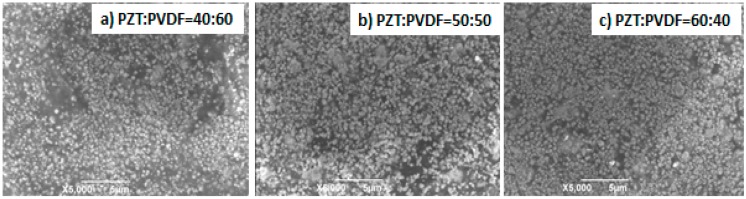
(**a**) The SEM images of the PZT/PVDF (40:60); (**b**) The SEM images of the PZT/PVDF (50:50); (**c**) The SEM images of the PZT/PVDF (60:40).

The SEM of PZT/PVC composite prepared by PVC with volume fractions of 40%, 50% and 60% respectively were shown in Figure 3. It can be seen from Figure 3, when the volume fraction of PZT was 40%, a large number of PZT particles were ‘suspended’ in the PVDF matrix, and some PVDF powder displays the reunion phenomenon. As the volume fraction of PZT increased to 50%, although some PZT powder was completely wrapped in the PVDF powder, most PZT particles were exposed to each other, and the PVDF powder was embedded in the gap between particles. When the volume fraction of PZT increases to 60%, the PZT particles are almost completely in contact with each other, and the distribution of PZT particles is very uniform.

**Figure 4:**
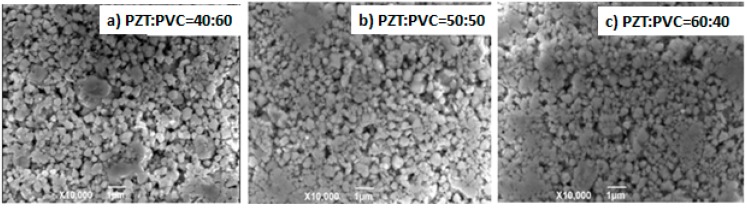
(**a**) The SEM images of the PZT/PVC (40:60); (**b**) The SEM images of the PZT/PVC (50:50); (**c**) The SEM images of the PZT/PVC (60:40).

Figure 4 is the SEM images of PZT/PVC composite prepared by PVC with the volume fractions of 40%, 50% and 60%, respectively. It can be seen from the Figure 4 that the PZT particles in composite materials are distributed evenly in the PVC matrix. With the increase of PZT content, the thickness of the PVC layer is reduced and the distance between PZT particles decreases. The interface combination between PZT and PVC is better, even the PZT content of PZT/PVC composite is high (PZT:PVC = 60:40), PVC is still tightly wrapped around the PZT particles. This is due to the PVC having satisfied bonding performance. The composites have dense matrix and a small number of pores, which is mainly caused by the PZT particle extraction process.

### 3.2. Effects of Polymer on the Dielectric Properties of PZT/Polymers

**Figure 5:**
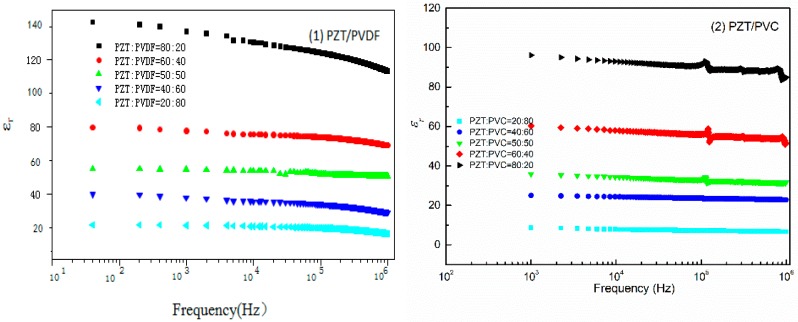
(**a**) Variation of dielectric constant of PZT/PVDF composites with frequency; (**b**) Variation of dielectric constant of PZT/PVC composites with frequency.

The frequency dependence of dielectric constant (ε_r_) of PZT/polymers was shown in Figure 5. The frequency stability of the PZT/PVDF composites is lower than that of the PZT/PVC composites. The dielectric constant of the PZT/PVDF composites and the PZT/PVC composites has the same trend as the change of the PZT volume content. The dielectric constant of PZT/polymer composites increased by increasing the addition of PZT, but the extent of the increase was different.

The dielectric properties of PZT/PVDF composites were higher than the dielectric properties of PZT/PVC composite with the same PZT contents. This indicates that the dielectric constant of the PZT/polymer composite materials is closely related to the polymer matrix’s own permittivity, and the polymer with high dielectric constant can obtain composite material with a high dielectric constant. When the PZT content of PZT/PVC composite reached 40%, and when PZT/PVDF composite reaches 50%, the curve shows a strong resonance peak, the reason is that the orientation polarization comes from the dipole of the PZT particles, the larger PZT content, the greater the polarization contribution and the more obvious the resonance peak.

**Figure 6:**
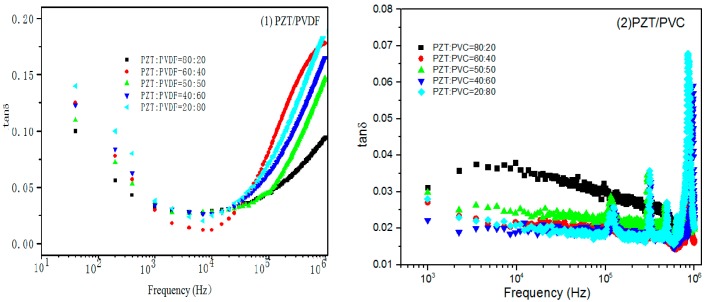
(**a**) Variation of dielectric loss of PZT/PVDF composites with frequency; (**b**) Variation of dielectric loss of PZT/PVC composites with frequency.

The relationship between the dielectric losses (tan δ) of two kinds of PZT/polymer composites is shown in Figure 6. It can be seen that, with the increase of frequency, the tan δ of both composite are slightly decreasing in the condition of low frequency (<10^4^ Hz), it is due to the relaxation of the interface charge. At high frequency (>10^4^ Hz), the tan δ of PZT/PVC change range differed greatly with the frequency increase but the values of all tan δ are small. In contrast with the increase of frequency the dielectric loss of the PZT/PVDF composites increases dramatically

At the same time, the dielectric loss of PVDF matrix composite is higher than that of PZT/PVC composite. The dielectric loss curves of PVDF and PVC matrix composite display the resonance peak, in particular, the PZT content of PZT/PVC composite has a loss peak when the PZT content is 40%, which is mainly due to the relaxation polarization caused by the dipole of PZT.

### 3.3. Effects of Polymer on the Piezoelectric Properties of PZT/Polymers

**Figure 7:**
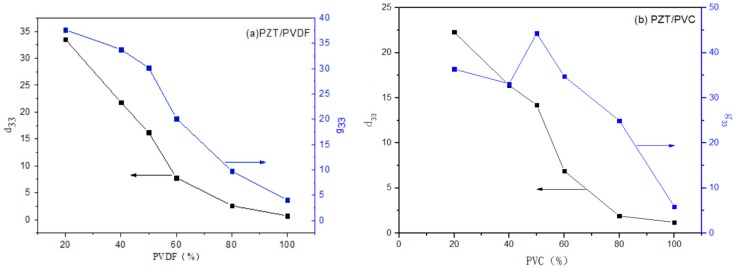
(**a**) Effect of PVDF volume fraction of the d_33_ of PZT/PVDF; (**b**) Effect of PVDF volume fraction of the g_33_ of PZT/PVDF.

As can be seen in Figure 7, with the increase of the PVDF volume fraction, the piezoelectric strain constant (d_33_) and the piezoelectric voltage constant (g_33_) of PZT/PVDF both presented decline gradually, this is because PVDF mainly played a role of a connection phase in PZT/PVDF, and PZT provided the high piezoelectric performance. Therefore, in order to obtain the piezoelectric properties and mechanical properties of the composite system, the volume fraction of PZT phase should not be less than 50%.

Figure 7b shows the relation curve between the piezoelectric properties of PZT/PVC composite prepared by PZT and different volume fraction of PZT. As can be seen in Figure 7b, with the increase of the volume fraction of PZT, the d_33_ and the g_33_ of the composites increase gradually, but when the volume fraction of the PZT is more than 50%, the d_33_ of the composites decreases with a certain amplitude, because when the PZT volume fraction is from 50% to 60%, the PVC particles of the composite material will form a coating around the ceramic layer. The results show that the piezoelectric properties of the composites show a decreasing trend when the content of PZT in ceramics exceeds 50%. Although the cohesive force between the two phases is increased, the charge transfer ability of the composites is reduced.

This is illustrated in the 0–3 composites, the electroactive particles are under-solicited because of the difference of dielectric constant between ceramics and polymers and because of the difference of elastic modulus [25]. When the volume fraction of the PZT is more than 60%, the piezoelectric properties of the PZT/PVDF increase with the increase of the PZT volume fraction, but are still lower than that of PZT/PVC composites.

## 4. Conclusions

(1) Two kinds of PZT/polymer composites were prepared by the hot pressing process using two thermoplastic polymers as the matrix. The results show that the polymer matrix has a great influence on the piezoelectric and dielectric properties of the PZT/polymer composites.
(2) The process for preparation of PZT/PVDF composite from semi-crystalline polymer PVDF was investigated. The piezoelectric properties of PZT/PVDF are the best when the PZT content is higher than 50%. The dielectric constant of PZT/PVDF composites is better than PZT/PVC, and the dielectric loss is slightly higher than that of the PZT/PVC when the frequency is 1 kHz.
(3) The PZT/PVC composites prepared by combining PZT with PVC can measure the resonance peak of the PZT when the content of PZT was 40%. The g_33_ of PZT/PVC composites is better than that of PZT/PVDF composites when the PZT content is 30–50%. The d_33_ of PZT/PVC composite is obviously better than that of PZT/PVDF composite. Therefore, the PZT/PVC composites will achieve high electromechanical coupling coefficients.
(4) Under the condition of frequency is 10^3^ Hz, the dielectric and piezoelectric properties of the PZT/PVC were both better than PZT/PVDF. When the volume fraction of PZT was 50%, the PZT/PVC composites prepared by the hot-press method could achieve excellent electrical performance. The ε_r_ was 65.75, the d_33_ was 16.2 Pc/N, the g_33_ was 28.26 mV·m/N, and the tan δ was only 0.037.

## References

[1] Gowdhaman P., Annamalai V., Thakur O.P. (2016). Piezo, ferro and dielectric properties of ceramic-polymer composites of 0–3 connectivity. Ferroelectrics.

[2] Li Q., Chen L., Gadinski M.R., Zhang S., Li H., Haque A., Jackson T., Wang Q. (2015). Flexible high-temperature dielectric materials from polymer nanocomposites. Nature.

[3] He C., Chen W.G. (2010). Preparation and application of piezoelectric materials and its research status. Funt. Mater..

[4] Ghosh M., Rao M.G. (2013). Growth mechanism of ZnO nanostructures for ultrahigh piezoelectric d_33_ coefficient. Mater. Express.

[5] KumaráThakur V., JináTan E., SeeáLee P. (2011). Surface functionalization of BaTiO_3_ nanoparticles and improved electricalal properties of BaTiO_3_/polyvinylidene fluoride composite. RSC Adv..

[6] Li H.T., Sun X.H. (2009). Research progress and development trend of piezoelectric ceramic materials. Adv. Ceram..

[7] Tiwari V., Srivastava G. (2005). Structural, dielectric and piezoelectric properties of 0–3 PZT/PVDF composite. Ceram. Int..

[8] Mendes S.F., Costa C.M., Sencadas V., Nunes J.S., Costa P., Grégório R. (2009). Effect of the ceramic grain size and concentration on the dynamical mechanical and dielectric behavior of poly(vinilidene fluoride)/Pb (Zr_0.53_Ti_0.47_) O_3_ composites. Appl. Phys. A.

[9] Zhang L.M., You D. (2004). The preparation and properties of 0–3 PZT/PVDF piezoelectric composites. Acta Mater. Compos. Sin..

[10] Li R., Pei J.Z. (2015). High dielectric performance of polyamide 11/poly(vinylidene fluoride) blend films induced by interfacial glycidyl methacrylate. Polym. Sci. Ser. A.

[11] Hu N., Liu X.N. (2008). Piezoelectric properties of 0–3 PZT/PVDF piezoelectric composites. J. Funt. Mater..

[12] Pardo L., Mendiola J., Alemany C. (1988). Theoretical treatment of ferroelectric composites using Monte Carlo calculations. J. Appl. Phys..

[13] Jaitanong N., Yimnirun R., Zeng H.R., Li G.R., Yin Q.R., Chaipanich A. (2014). Piezoelectric properties of cement based/PVDF/PZT composites. Mater. Lett..

[14] Ahmad Z., Prasad A., Prasad K. (2009). A comparative approach to predicting effective dielectric, piezoelectric and elastic properties of PZT/PVDF composites. Physica B.

[15] Ende D.A., Almeida P.D., Zwaag S.V.D. (2007). Piezoelectric and mechanical properties of novel composites of PZT and a liquid crystalline thermosetting resin. J. Mater. Sci..

[16] Olhero S.M., Garcia G.L., Button T.W., Alves F.J., Ferreira J.M. (2012). Innovative fabrication of PZT pillar arrays by a colloidal approach. J. Eur. Ceram. Soc..

[17] Nan C.W., Birringer R., Clarke D.R., Gleiter H. (1997). Effective thermal conductivity of particulate composites with interfacial thermal resistance. J. Appl. Phys..

[18] Li R., Wang H.F., Liu H.J., Pei J.Z. (2016). Influence of PZT piezoelectric ceramics on the structure and electric properties of PZT/poly(vinylidene fluoride)composites. Mater. Express.

[19] Sulaiman M., Rahman A.A., Mohamed N.S. (2015). Effect of water-based sol-gel method on structural, thermal and conductivity properties of LiNO_3_–Al_2_O_3_ composite solid electrolytes. Arab. J. Chem..

[20] Bai W., Yang D.B. (2011). Dielectric and piezoelectric properties of 0–3 composite film in PCM/PVDF and PZT/PVDF. Ferroelectrics.

[21] Zak A.K., Gan W.C., Majid W.H. (2011). Experimental and theoretical dielectric studies of PVDF/PZT nanocomposite thin films. Ceram. Int..

[22] Guan X., Zhang Y., Li H., Ou J. (2013). PZT/PVDF composites doped with carbon nanotubes. Sens. Actuators A.

[23] Gregorio R., Cestari M., Bernardino F.E. (1996). Dielectric behaviour of thin films of β-PVDF/PZT and β-PVDF/BaTiO3 composites. J. Mater. Sci..

[24] Martins P., Lopes A., Lanceros-Mendez S. (2014). Electroactive phases of poly (vinylidene fluoride): Determination, processing and applications. Prog. Polym. Sci..

[25] Defebvin J., Barrau S., Lyskawa J., Woisel P., Lefebvre J.M. (2017). Influence of nitrodopamine functionalized barium titanate content on the piezoelectric response of poly(vinylidene fluoride) based polymer-ceramic composites. Compos. Sci. Technol..

